# Analysis of *C. elegans* NR2E nuclear receptors defines three conserved clades and ligand-independent functions

**DOI:** 10.1186/1471-2148-12-81

**Published:** 2012-06-12

**Authors:** Katherine P Weber, Christopher G Alvaro, G Michael Baer, Kristy Reinert, Genevieve Cheng, Sheila Clever, Bruce Wightman

**Affiliations:** 1 Biology Department, Muhlenberg College, Allentown, PA, 18104, USA

## Abstract

**Background:**

The nuclear receptors (NRs) are an important class of transcription factors that are conserved across animal phyla. Canonical NRs consist of a DNA-binding domain (DBD) and ligand-binding domain (LBD). While most animals have 20–40 NRs, nematodes of the genus *Caenorhabditis* have experienced a spectacular proliferation and divergence of NR genes. The LBDs of evolutionarily-conserved *Caenorhabditis* NRs have diverged sharply from their *Drosophila* and vertebrate orthologs, while the DBDs have been strongly conserved. The NR2E family of NRs play critical roles in development, especially in the nervous system. In this study, we explore the phylogenetics and function of the NR2E family of *Caenorhabditis elegans*, using an *in vivo* assay to test LBD function.

**Results:**

Phylogenetic analysis reveals that the NR2E family of NRs consists of three broadly-conserved clades of orthologous NRs. In *C. elegans*, these clades are defined by *nhr-67, fax-1* and *nhr-239.* The vertebrate orthologs of *nhr-67* and *fax-1* are *Tlx* and *PNR,* respectively. While the *nhr-239* clade includes orthologs in insects (*Hr83*), an echinoderm, and a hemichordate, the gene appears to have been lost from vertebrate lineages. The *C. elegans* and *C. briggsae nhr-239* genes have an apparently-truncated and highly-diverged LBD region. An additional *C. elegans* NR2E gene, *nhr-111*, appears to be a recently-evolved paralog of *fax-1;* it is present in *C. elegans*, but not *C. briggsae* or other animals with completely-sequenced genomes. Analysis of the relatively unstudied *nhr-111* and *nhr-239* genes demonstrates that they are both expressed—*nhr-111* very broadly and *nhr-239* in a small subset of neurons. Analysis of the FAX-1 LBD in an *in vivo* assay revealed that it is not required for at least some developmental functions.

**Conclusions:**

Our analysis supports three conserved clades of NR2E receptors, only two of which are represented in vertebrates, indicating three ancestral NR2E genes in the urbilateria. The lack of a requirement for a FAX-1 LBD suggests that the relatively high level of sequence divergence for *Caenorhabditis* LBDs reflects relaxed selection on the primary sequence as opposed to divergent positive selection. This observation is consistent with a model in which divergence of some *Caenorhabditis* LBDs is allowed, at least in part, by the absence of a ligand requirement.

## Background

The nuclear receptors (NRs) constitute a class of transcriptional regulators that are conserved throughout the animal kingdom, where they function in a wide variety of physiological and developmental roles, including metabolic regulation, xenobiotic defense, and development [1-4]. Archetypal NRs have a DNA-binding domain (DBD), which contains two C_4_ zinc fingers and mediates binding to specific DNA sequences and receptor dimerization, and a more C-terminal ligand-binding domain (LBD), which may bind a lipophilic ligand and functions in dimerization, nuclear localization, and transcriptional trans-activation. Across the animal kingdom, amino acid sequence similarity is strongly conserved in the DBD, and more weakly conserved in the LBD. The majority of NRs that have been identified on the basis of phylogenetic sequence relationship have no known ligand, despite having a recognizable LBD and are sometimes referred to as “orphan receptors” [1].

The NR superfamily has proliferated and diverged to a striking degree in the nematode *Caenorhabditis elegans*: while the *C. elegans* genome boasts 284 predicted NRs, the human genome has only 48 and the *Drosophila melanogaster* genome only 21 [5-8]. Only 15–20 *C. elegans* NRs are clearly orthologous to NRs that are broadly conserved among animal phyla [8]. The remaining (approximately 265) *C. elegans* predicted NRs appear to have evolved from an HNF4 ancestor and do not have clear orthologs in non-nematodes or distantly-related nematodes, suggesting that these genes have proliferated within the nematode phylum [5,9,10]. Nematode species that are closely related to *C. elegans* display a similar expansion of the NR gene family. *Caenorhabditis briggsae* and *Caenorhabditis remanei*, two species that are separated from *C. elegans* by about 100 million years and are morphologically indistinguishable from *C. elegans*, contain 232 and 256 NR genes, respectively [11]. Furthermore, only about half of *C. elegans* NRs are conserved in both *C. briggsae* and *C. remanei* as three-way orthologs, while an additional 10% are present as two-way orthologs [12]. Thus, the rapid expansion of the NR family appears to have begun before the separation of *C. elegansC. briggsae* and *C. remanei*, and continued separately in all three species. The more distantly related nematode *Pristionchus pacificus* also has an expanded complement of 167 nuclear receptors [13,14], (R. Sommer, personal communication). The driving evolutionary explanation for this proliferation is unclear, but it could reflect adaptation to the complex chemical and xenobiotic environment of soil life [5-8]. In contrast, this expansion is not present in the genome of the nematode *Brugia malayi*, a human parasite: it contains only 27 NR genes, most of which are conserved across animal phyla [15]. Therefore, evolution of NRs is unexpectedly dynamic among free-living nematodes, suggesting that proliferation and divergence in the NR family may play a major role in nematode speciation. This possibility elevates the importance of understanding NR evolution and function in nematodes.

One explanation for the wide variety of *Caenorhabditis* NRs and the radiating divergence of LBD sequences is a lack of hormone-responsiveness. NRs that are known to bind ligands in other invertebrates, such as the ecdysone receptor, are absent in *Caenorhabditis*[5]. However, a bona fide ligand has been identified for the *C. elegans* NR DAF-12 [16], and most *C. elegans* NRs have a recognizable LBD, raising the possibility that other *C. elegans* NRs may also bind as yet unknown ligands. Even if some *Caenorhabditis* NRs are not hormone-responsive, the LBD may be retained for its other functions, such as transcriptional modulation, receptor dimerization, and/or nuclear localization. In any of these cases, the wide variety of *Caenorhabditis* LBD sequences could reflect a wider diversity of inputs or outputs, or a relaxation of selection on LBD primary sequences.

The nuclear receptors have been grouped into phylogenetically conserved families on the basis of amino acid sequence relationship [7,17]. Members of the NR2E group include *tailless (tll)* and *unfulfilled (unf)* in *DrosophilaTlx* and *PNR* in vertebrates, and *nhr-67* and *fax-1* in *C. elegans*. These NRs have been systematically defined as NR2E1 through NR2E5. Another family member, designated NR2E6, has been identified in insects [18]. The NR2E nuclear receptors that have been functionally characterized have a common theme of function in nervous system development. While mutations in the *tll* gene of *Drosophila* were first identified based on their disruption of anterior-posterior patterning, subsequent analysis demonstrated functions in embryonic CNS and larval eye development [19,20]. The mouse *Tlx* gene functions in limbic system and eye development, but is not required for overall patterning of the embryo [21-23]. *C. elegans fax-1*, its vertebrate homolog *PNR*, and *Drosophila* homolog *unf* all play key roles in regulating neuron development [24-29]. The NR2E class is of particular importance from an evolutionary perspective as well; it includes members from the cnidaria to vertebrates, indicating that it is of ancient evolutionary ancestry [4]. DNA-binding studies suggest that DBD properties of FAX-1 and NHR-67 are at least partially evolutionarily-conserved [30]. However, like other *C. elegans* LBDs, the NR2E class LBDs are more highly-diverged from each other and from orthologous LBDs.

We have studied the conserved NR2E class of NRs in *C. elegans* to examine how the low level of LBD sequence conservation affects function using an *in vivo* functional assay. Our analysis of *C. elegans* NR2E-related sequences identified one unstudied “satellite” nuclear receptor, *nhr-111*, which is related to *fax-1,* but is not present in any other genome sequenced to date, and another NR, *nhr-239*, which is a member of new, relatively uncharacterized clade of conserved NRs found in non-vertebrate animals that includes insect *Hr83*. Using an *in vivo* functional assay, we tested whether LBD function was required for *fax-*1 and if LBD functions of other NR2E genes could be substituted for the *fax-1* LBD function.

## Results

### **NR2E family receptors can be grouped into three major conserved clades**

Systematic classifications of NRs have grouped vertebrate Tlx-like genes into an NR2E1 subgroup, insect TLL-like genes into an NR2E2 subgroup, vertebrate PNR-like genes into an NR2E3 subgroup, and *C. elegans* FAX-1 as an NR2E5 subgroup [17,31,32]. Additional genes were designated NR2E4 and NR2E6, but these appear to be absent from both nematodes and vertebrates and are not considered further in this analysis. NR2E1 and NR2E2 subfamily members have very similar DBDs and DNA-binding activities, as do NR2E3 and NR2E5 subfamily members [30,33,34]. Ecdysozoans have an NR2E2, but not an NR2E1, and vice versa for vertebrates. The simplest explanation for this relationship is the existence of a single common ancestral gene for NR2E1/NR2E2. A similar scenario is likely for the NR2E3/NR2E5 group.

**Figure 1:**
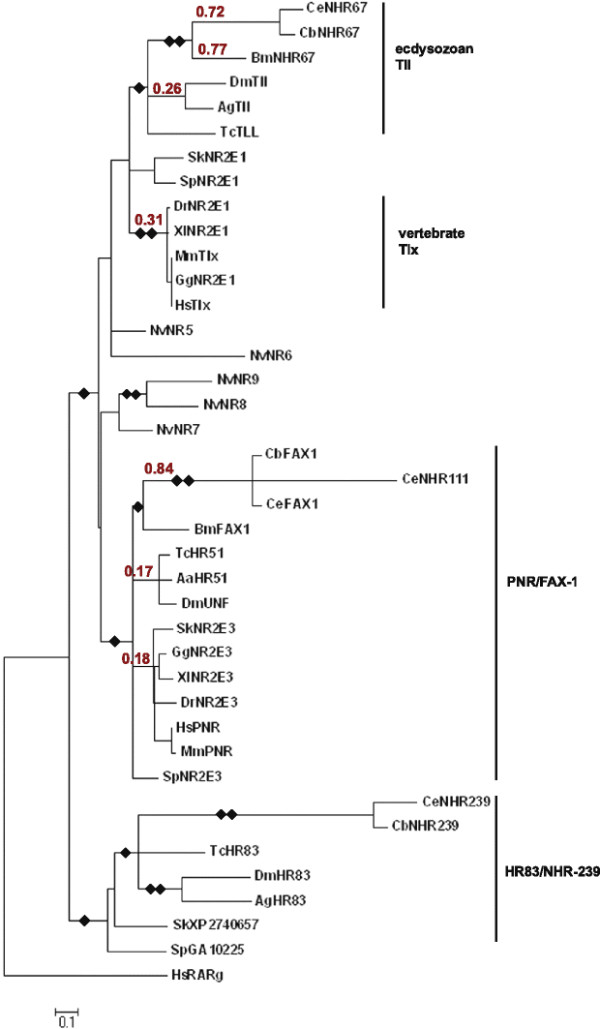
**Phylogenetic analysis of NR2E NRs.** Maximum Likelihood trees of ClustalW-aligned amino acid sequences were calculated using MEGA 5.0 [35,36]. Highly-divergent regions N-terminal to the DBD and C-terminal to the LBD were not included. A consensus tree generated from 500 bootstrap replicates and rooted to the *Homo sapiens* retinoic acid receptor gamma outgroup sequence is shown. Further details are provided in Methods. Species abbreviations (used throughout): Ce (or no annotation)*:Caenorhabditis elegans*, Cb*: Caenorhabditis briggsae,* Bm: *Brugia malayi*, Pp: *Pristionchus pacificus,* Dm: *Drosophila melanogaster*, Ag: *Anopheles gambiae*, Tc: *Tribolium castaneum*, Sk: *Saccoglossus kowalevskii*, Sp: *Strongylocentrotus purpuratus*, Dr: *Danio rerio*, Xl: *Xenopus laevis*, Mm: *Mus musculus*, Gg: *Gallus gallus*, Hs: *Homo sapiens*, Nv: *Nematostella vectensis*, Aa: *Aedes aegypti*. Tree is proportionally scaled, with the scale bar indicating sequence distance in units of substitutions. Single diamonds identify branchpoints that were supported by >50 % of bootstrap replicates; double diamonds identify branchpoints that were supported by >95 % of bootstrap replicates. Numbers in red above selected branches show Ka/Ks ratios for the LBD portion only of NRs.

The *C. elegans* genome project identified the NR2E-like nuclear receptor *nhr-239* after the publication of a broad phylogenetic analysis to resolve evolution of the NR superfamily [32]. In order to determine the phylogenetic relationship of *nhr-239* to other nematode and non-nematode nuclear receptors, we performed Clustal W multiple sequence alignment and constructed trees using Maximum Likelihood and Neighbor-Joining methods with the predicted *C. elegans* NHR-239 protein sequence. In addition to trees based on aligned DBD and LBD sequences (Figure 1), we also evaluated trees using the DBD alone, since substitution rates in *Caenorhabditis* LBDs are much higher than in other species, which would lead to artificially inflated branch lengths for full-length *Caenorhabditis* NRs. We included nuclear receptors from complete or nearly complete genome projects, including ecdysozoans (*C. elegans, C. briggsae, Pristionchus pacificus, Brugia malayi, Aedes aegypti, Anopheles gambiae, Drosophila melanogaster,* and *Tribolium castaneum*), vertebrates (*Homo sapiens, Mus musculus, Gallus gallus, Xenopus laevis,* and *Danio rerio*), a hemichordate (*Saccoglossus kowalevskii*), an echinoderm (*Strongylocentrotus purpuratus*), and a cnidarian (*Nematostella vectensis*). Maximum Likelihood and Neighbor-Joining computational methods produced similar results. *C. elegans* NHR-239 was organized into a diverged NR2E-like clade that includes relatively unstudied nuclear receptors from invertebrates, including the *Drosophila* nuclear receptor Hr83 (Figure 1). Because this group includes genes from deuterostomes (*Saccoglossus* and *Strongylocentrotus*), we infer that the NHR-239/Hr83 clade derives from a gene that was present in the urbilaterian ancestor, but has subsequently been lost from vertebrate lineages.

Phylogenetic trees with full-length NR2E sequences also identified a highly-supported PNR/FAX-1 clade, with ecdysozoan clade members on one branch and deuterostome clade members on a separate branch (Figure 1). As observed in earlier phylogenetics analyses [32], a single TLX/TLL clade was not supported, with ecdysozoan, hemichordate, and vertebrate clade members occupying separate unresolved branches. Analysis of the DBD alone, however, organized vertebrate TLX and ecdysozoan TLL members into a single clade (Additional file 1: Figure S1). The *C. elegans* FAX-1 DBD very closely resembled those of *D. melanogaster* UNF (92.9% identical) and *H. sapiens* PNR (83.3% identical), but it was much less similar to the *D. melanogaster* Hr83 DBD (64.3% identical; Table 1; Additional file 1: Figure S1). In contrast, the *C. elegans* NHR-239 DBD was more similar to *D. melanogaster* Hr83 than to DmUNF (60.7% vs. 57.1%). Taken together, we interpret these data to define three major conserved clades of NR2E NRs: a TLX/TLL clade, a PNR/FAX-1 clade, and an NHR-239/Hr83 clade.

**Table 1:** Percent identities between NRs by domain

	**% IDENTICAL**	
	**DBD**	**LBD**
**CeFAX-1 compared to:**		
CbFAX-1	100.0 %	69.0 %
BmFAX-1	94.0 %	17.2 %
PpFAX-1	93.0 %	10.4 %
DmUNF	92.9 %	17.3 %
HsPNR	83.3 %	16.4 %
CeNHR-67	65.5 %	12.7 %
HsTLX	66.7 %	15.5 %
CeNHR-111	57.1 %	25.0 %
DmHR83	64.3 %	16.4 %
**DmUNF compared to:**		
BmFAX-1	89.3 %	45.9 %
PpFAX-1	86.0 %	12.1 %
HsPNR	83.3 %	50.8 %
**CeNHR-67 compared to:**		
CbNHR-67	95.2 %	67.2 %
BmNHR-67	89.3 %	27.3 %
PpNHR-67	88.5 %	N/A
DmTLL	84.5 %	18.5 %
HsTLX	76.2 %	18.1 %
**DmTLL compared to:**		
BmNHR-67	84.5 %	21.4 %
PpNHR-67	79.3 %	N/A
HsTLX	82.1 %	42.9 %
**CeNHR-239 compared to:**		
CbNHR-239	90.5 %	54.9 %
PpNHR-239	68.7 %	N/A
DmHR83	60.7 %	8.5 %
SkXP2740657	64.3 %	8.6 %
DmUNF	57.1 %	8.5 %

Amino acid sequences were aligned by ClustalW and evaluated using MEGA 5.0 software [35,36]. Figures shown are the percent identical at each amino acid position in pairwise comparison, with unaligned regions deleted. Species abbreviations are given in Figure 1.

Previous analysis and annotation of NR2E NRs did not benefit from complete data sets and were not focused on the NR2E group in particular. Two phylogenetic analyses of NRs that did not include NHR-239 grouped *C. elegans* FAX-1 with *Drosophila* Hr83 [32,34]. These analyses were performed with phyletically-broad datasets using full-length NRs. When *C. elegans* NRs are included, the relatively high divergence of the LBD tends to pull *C. elegans* branches out of clades, leading to the possibility for long-branch attraction to weakly assemble *C. elegans* NRs into clades to which they do not belong [37]. This has led to an annotation problem where some members of the NHR-239/Hr83 clade, including Hr83 itself, have been designated “Fax1” and received an NR2E5 systematic designation (Table 2). The inclusion of our larger set of NHR-239/Hr83 data argues that this annotation is misleading and should be revised.

**Table 2:** Annotation of NR2E orthologs by clade

**Human**	***C. elegans***	***Drosophila***	**Other species**					
*Tlx*	*nhr-67*	*tailless*	*Nematostella* NvR5 (2)					
[NM 003269]	[NM 069693]	[NM 079857]	*--------------------------------------------------------------------------------------------------------------------------------------------------------------------------------------------------------------------------------------------------------------------------*					
			*Apis* AmTll (3)					
			*Bombyx* Tll (4)					
			*Tribolium* Tll (5)					
			*Brugia* BmNHR-15 (6)					
			*Schmidtea* Tlx (7)					
			*--------------------------------------------------------------------------------------------------------------------------------------------------------------------------------------------------------------------------------------------------------------------------*					
			*Strongylocentrotus* Tll (8)					
			*--------------------------------------------------------------------------------------------------------------------------------------------------------------------------------------------------------------------------------------------------------------------------*					
			*Saccoglossus* NP 001158362;Tll (9)					
*PNR*	*fax-1*	*unfulfilled/*	*Nematostella* NvR6-NvR9 (2)					
[NM 016346]	[NM 076146]	*Hr51*	*--------------------------------------------------------------------------------------------------------------------------------------------------------------------------------------------------------------------------------------------------------------------------*					
		[NM 137188]	*Apis* AmHr51 (3)					
			*Bombyx* BmHr51 (4)					
			*Tribolium* Hr51 (5)					
			*Brugia* BmNHR-16 (6)					
			*--------------------------------------------------------------------------------------------------------------------------------------------------------------------------------------------------------------------------------------------------------------------------*					
			*Strongylocentrotus* Pnr (8)					
			*--------------------------------------------------------------------------------------------------------------------------------------------------------------------------------------------------------------------------------------------------------------------------*					
			*Saccoglossus* NP 001158447; PNR (9)					
No ortholog	*nhr-239*	*Hr83* (1)	*Nematostella* NvR6-NvR9 (2)					
	[NM 065178]	[NM 141390]	*--------------------------------------------------------------------------------------------------------------------------------------------------------------------------------------------------------------------------------------------------------------------------*					
			*Apis* AmHr83 (3)					
			*Bombyx* ortholog not identified (4)					
			*Tribolium* Hr83 (5)					
			*Brugia* BmNHR-C* (6)					
			*--------------------------------------------------------------------------------------------------------------------------------------------------------------------------------------------------------------------------------------------------------------------------*					
			*Strongylocentrotus* “Fax1” (8)					
			*--------------------------------------------------------------------------------------------------------------------------------------------------------------------------------------------------------------------------------------------------------------------------*					
			*Saccoglossus* XM 002740611; “NR6A1-like” (9)					

(1) Annotated as *Drosophila* “Fax-1” in [32].

(2) *Nematostella vectensis*, four NR2E orthologs not clearly resolved, [38], this work.

(3) *Apis mellifera*[39].

(4) *Bombyx mori*[40].

(5) *Tribolium castaneum*[18,41].

(6) *Brugia malayi*[15].

(7) *Schmidtea mediterranea*[42].

(8) *Strongylocentrotus purpuratus*[43].

(9) *Saccoglossus kowalevskii*[44,45], this work.

* Incomplete gene assembly, tentative gene designation. The “Other species” column is organized by taxonomic groups: cnidarian, ecdysozoa, echinoderm, and hemichordate.

**Figure 2:**
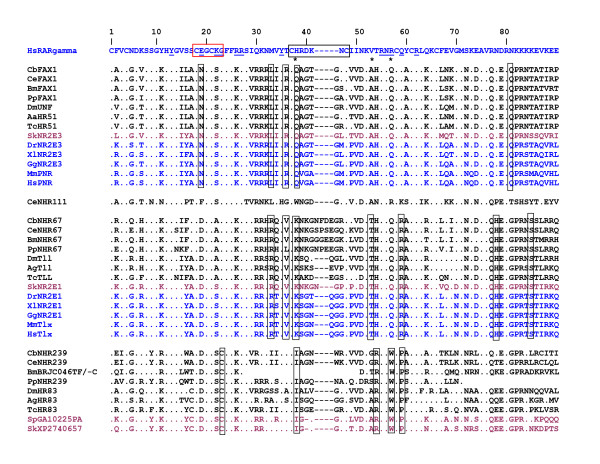
**Alignments of NR2E DBDs.** Annotated alignments of NR2E DBDs based on ClustalW multiple alignments as described in Methods. The reference sequence of the human retinoic acid receptor gamma subunit is shown at the top. The red box identifies the P box, which plays a critical role in DNA binding site specificity. The blue box identifies the D box, which functions in protein dimerization. Underlined amino acid positions identify points of contact between RAR/RXR heterodimers and DNA bases, and asterisks identify protein dimerization contacts in structural studies [46]. Color scheme distinguishes ecdysozoans (black), non-vertebrate deuterostomes (red), and vertebrates (blue). Vertical boxes identify amino acids common to a particular clade (TLL/TLX, PNR/FAX-1, Hr83/NHR239). Species abbreviations are given in Figure 1. The *Brugia malayi* sequence is derived from separate candidate coding sequences that have not been assembled into a gene model or confirmed as an expressed gene. The C-terminal portion of the predicted *Pristionchus pacificus* NHR-239 ortholog was not included since it aligned very poorly and it is unclear whether this reflects true divergence, incorrect gene assembly, or a pseudogene. It is also possible that the *B. malayi* ortholog shown here is actually a pseudogene, since it has not been confirmed by a cDNA.

Analysis of aligned DBD sequences also revealed amino acids at certain positions that were diagnostic for each of three clades (Figure 2). Analysis of the PNR/FAX-1 clade identifies six amino acid positions in the DBD that are identical among all members of this clade, including Asn-19, which plays a key role in DNA-binding specificity [30], and Gln-38, which is predicted to mediate protein dimerization contacts [46]. The TLX/TLL and NHR-239/Hr83 clades have a conserved Asp-19, suggesting some possible overlap in DNA-binding specificity. The TLX/TLL group has seven clade-specific positions, including the potentially important Lys-38 [46]. All three nematode TLX/TLL clade members (NHR-67) have a D box that is expanded by an insertion of four amino acids (relative to most NRs) as compared to a single D box amino acid insertion for TLX and TLL proteins from non-nematodes (Figure 2). This suggests that dimerization properties of nematode NRs may have diverged from other clade members. Finally, the NHR-239/Hr83 group has five clade-specific positions, including Ile-38 and Trp-57 (which is a statistically infrequent amino acid [47]), both of which are positions predicted to be involved in DNA-binding and dimerization (Figure 2). The coordinated conservation of these residues may reveal functionally-significant positions that help determine specificity, and supports the organization of these clades as representing distinct evolutionary origins.

As expected, the * Caenorhabditis * NR2E LBDs aligned more poorly than the DBDs with insect and vertebrate orthologs (Table 1; Additional file 2: Figure S2). Ka/Ks ratios for LBD regions (Figure 1) at branches that lead to nematode NRs were high (0.72 to 0.84), in comparison to branches that lead to insect or vertebrate NRs (0.17 to 0.31). In contrast, Ka/Ks ratios for DBD regions (Additional file 1: Figure S1) were relatively low at nematode and non-nematode branches (0.04 to 0.21 for the more strongly-conserved PNR/FAX-1 and TLX/TLL clades). These data suggest strong purifying selection on the DBD across phyletic groups and very weak purifying selection on nematode LBDs. Furthermore, some key positions in the LBD signature domain and other regions that are conserved in insects and vertebrates have diverged in *Caenorhabditis* (Additional file 2: Figure S2). The *Brugia malayi* orthologs of FAX-1 and NHR-67 seem to have followed different evolutionary histories. While the LBD of *Brugia* FAX-1 looks similar to *Drosophila* UNF (45.9% identical) and human PNR (50.8%), the LBD of *B. malayi* NHR-67 looks more similar to *C. elegans* NHR-67 (27.3% identical) than to *D. melanogaster* (21.4%; Table 1). Therefore, the *B. malayi* FAX-1 LBD appears to have followed an insect-like path, while the *B. malayi* NHR-67 LBD appears to have followed a free-living nematode-like path. The NHR-239/Hr83 clade LBDs are considerably more diverged from each other and the LBDs of other clades (Table 1; Additional file 2: Figure S2). This was particularly true for *C. elegans* and *C. briggsae* NHR-239, which align very poorly in the LBD signature region with other NR2E LBDs and are apparently truncated (Additional file 2: Figure S2). This observation suggests that *Caenorhabditis* NHR-239 may lack a bona fide LBD, like *C. elegans* ODR-7 and *Drosophila* KNIRPS [48,49].

Analysis of NR2E nuclear receptors of the nematode *Pristionchus pacificus* suggested extensive sequence divergence of LBDs in this species. While the predicted *P. pacificus* FAX-1 ortholog aligned well with PNR/FAX-1 clade DBDs (Figure. 2; Table 1), the predicted LBD aligned very poorly (Table 1; Additional file 2: Figure S2). In particular, key features of the LBD signature domain that were present in other nematode LBDs were not found in the *P. pacificus* FAX-1 LBD. Analysis of *P. pacificus* genomic sequence also identified candidate NHR-67 and NHR-239 orthologs by conservation of DBD sequences (Figure 2; Table 1), but failed to identify coding sequences or gene assembly models with similarity to TLX/TLL and NHR-239/Hr83 clade LBDs (data not shown). The *P. pacificus* genomic sequence is mostly complete [13], however gene assembly and confirmation by cDNA analysis is not yet comprehensive. Nonetheless, the absence of LBD sequences with a clear relationship to *Caenorhabditis* or *Brugia* NR2E LBDs suggests that LBD divergence of *P. pacificus* NR2E LBDs may be much greater than that observed in *Caenorhabditis*. This conclusion contrasts with the relatively strong conservation of LBD sequences reported for *P. pacificus* RXR (NR2B) and ecdysone (NR1H) receptors [50].

The *C. elegans* genome project also identified another potential NR2E gene family member, *nhr-111*. No clear ortholog of *nhr-111* has been identified in any other species, including the fully-sequenced and closely-related *C. briggsae* and *C. brenneri* nematodes. The NHR-111 sequence is included in the PNR/FAX-1 clade when full-length proteins are considered (Figure. 1), but is not included when the DBD alone is considered (Additional file 1: Figure S1). Alignment of the NHR-111 DBD revealed divergence of the NHR-111 DBD from other NR2E family members at multiple positions (Table 1; Figure 2). However, the NHR-111 DBD was still more similar to NR2E DBDs than to the DBDs of other nematode NRs (data not shown). Inclusion of NHR-111 in the full-length NR2E clade occurred due to extensive similarity between the NHR-111 LBD and FAX-1 LBD (25% identical in Table 1; Additional file 2: Figure S2). From these observations, we propose that the *nhr-111* gene arose from a relatively recent duplication of the *fax-1* gene in the *C. elegans* evolutionary lineage, followed by extensive divergence of the DBD.

### **The novel* nhr-111 *and* nhr-239 *NRs are expressed genes**

**Figure 3:**
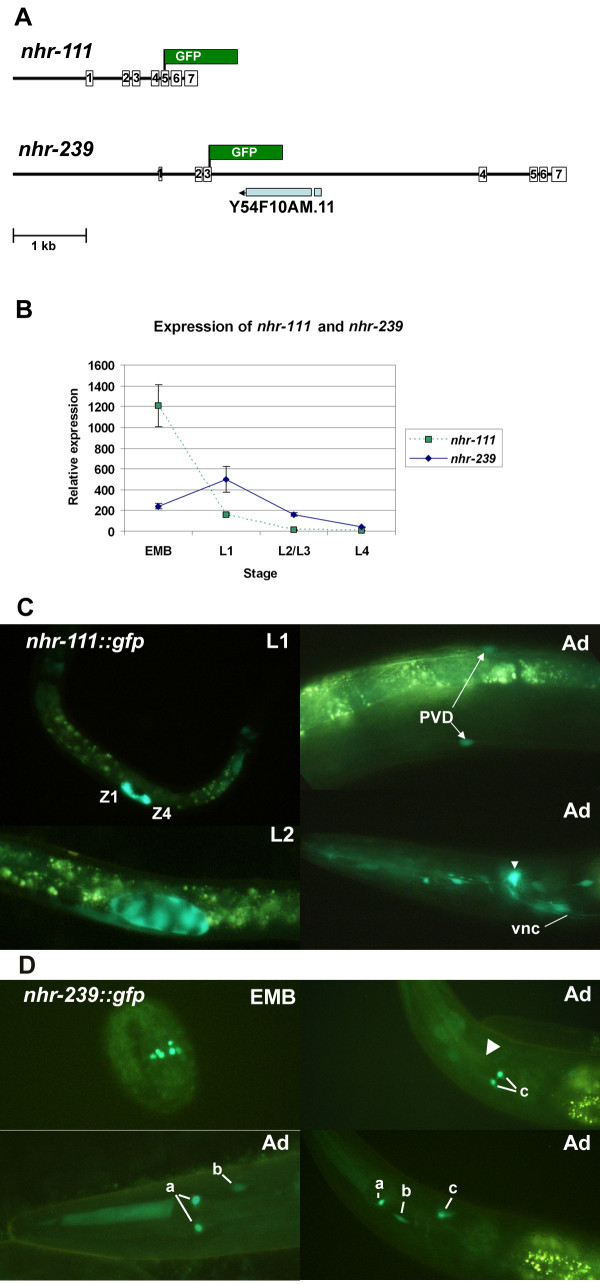
*** C. elegans nhr-111 *and *nhr-239 *are expressed genes.** The *nhr-111* and *nhr-239* genes and their expression patterns are shown. **A.** Schematic of the *nhr-111* and *nhr-239* genes showing the basic gene structure and point of fusion to GFP in expression constructs. **B.** Expression of the *nhr-111* and *nhr-239* genes over time as measured in qRT-PCR experiments. Arbitrary expression units are 2^-ΔCt^x10^10^. Error bars represent the standard deviation among replicates. Data from embryos (EMB) and larval L1, L2/L3, and L4 preparations of wild-type cDNA are shown. **C.** Expression of the *nhr-111::gfp* transgene in L1, L2 and adult (Ad) animals. The Z1 and Z4 gonad precursors in the L1 are labelled. The L2 image shows expression throughout the developing somatic gonad. The PVD neurons and ventral nerve cord (vnc) are indicated in the adult panels. Arrowhead identifies the location of a prominent dorsal neuron that appears to be sensory. **D.** Expression of the *nhr-239::gfp* transgene in a two-fold embryo (EMB) and adult (Ad) hermaphrodites. Individual neuron pairs are identified by lower case letters: a, pair of pharyngeal neurons; b, pair of mid-lateral neurons or neuronal support cells; c, pair of dorsal neurons just posterior to the nerve ring with sensory dendritic processes (arrowhead). Due to the low level of *nhr-239::gfp* expression, brightness of the images in D was significantly increased relative to those in C.

While *fax-1* and *nhr-67* have been studied in some detail, the *nhr-111* and *nhr-239* genes have not been characterized. Therefore, we considered the possibility that *nhr-111* or *nhr-239* could represent pseudogenes. While cDNA clones have been identified for *nhr-111*, spliced *nhr-239* products have only been identified by deep sequencing (http://www.wormbase.org), suggesting that the latter transcript may be very rare. We used nested PCR to identify a partial *nhr-239* cDNA from the 3’ end of the gene, which confirms a portion of the predicted and deep-sequencing reported exon-intron structure (Figure 3A). We used real-time qRT-PCR to examine the temporal dynamics of *nhr-111* and *nhr-239* expression (Figure 3B). *nhr-111* transcripts were detected at modest levels in embryos, but decreased progressively during larval development. *nhr-239* transcripts were identified at very low levels (no more than 2x10^-6^ the level of 18 S rRNA standards) and displayed a more complicated pattern, increasing somewhat from embryos to L1, before declining progressively during later larval development.

In order to examine cell-specific expression of both genes in living nematodes, we constructed *nhr-111::gfp* and *nhr-239::gfp* transgenes (Figure 3A). The *nhr-111::gfp* reporter was consistently expressed in at least eight pairs of neurons in the head, the sensory PVD neurons of the posterior lateral body wall, the pharynx, intestine (most often in the posterior- and anterior- most cells), the dorsal peri-vulva region of adults (which may be either uterine or vulval cells), and the somatic gonad precursor cells (Figure 3C). Among the head neurons was one prominent pair of sensory neurons just posterior to the nerve ring and at least one pair of neurons or support cells that appear to be inner or outer labial sensory cells. We also observed weak and variable expression in a subset of ventral nerve cord motorneurons. The temporal dynamics of *nhr-111::gfp* were consistent with *nhr-111* qRT-PCR results: expression was very bright in the Z1 and Z4 somatic gonad precursor cells in embryos and early L1, but decreased in the developing gonad at later stages and was relatively faint in other cells. Therefore, despite its relatively recent evolutionary origin, the *nhr-111* gene is fairly broadly expressed.

The *nhr-239::gfp* reporter was weakly expressed in three to four pairs of neurons in the head and a pharyngeal neuron in late stage embryos and all larval and adult stages (Figure 3D). One pair of dorsal neurons express *nhr-239::gfp* very consistently and appear to be sensory, as do one pair of pharyngeal cells that appear to be the MC, NSM or M3 neurons. We observed faint fluorescence in the pharynx (which may be an artefact), but did not observe *nhr-239* expression in other cells at any stage. As expected from the modest expression levels observed in qRT-PCR experiments, fluorescence from the *nhr-239::gfp* transgene was very faint. While it is possible that our translational fusion did not recapitulate the entire *nhr-239* expression pattern, this result suggests that *nhr-239* is expressed only in a very limited subset of neurons. Taken together, these data demonstrate that both genes are expressed, and that both may play roles in neuron development like other NR2E family members.

### **FAX-1, NHR-67, and NHR-111 have predicted LBD structures that are similar to defined vertebrate LBDs**

Like most *C. elegans* NRs, FAX-1, NHR-67, and NHR-111 proteins have extended C-terminal regions after the DBD that have some features of canonical NR LBDs (Additional file 2: Figure S2). However, the greater divergence at the level of primary sequence makes diagnosis of a probable LBD more difficult in *C. elegans* than in *Drosophila* and other animals. In the case of NHR-239, the C-terminal region is truncated and much more highly diverged in both *C. elegans* and *C. briggsae*, suggesting that this NR has dispensed with a bona fide LBD. Lipophilic ligands generally bind internally to LBDs, raising the possibility that for NR LBDs, structure may be a more important consideration than primary sequence [51]. To examine the structural capacity for *C. elegans* NR2E C-terminal regions to encode LBDs, we used PSIPRED and the threading utility pGenTHREADER. This application compares predicted secondary structures derived from primary protein sequence to a database of solved tertiary structures to predict likelihood of structural match [52,53]. The *C. elegans* FAX-1, *C. briggsae* FAX-1, and *C. elegans* NHR-111 and NHR-67 LBDs all returned top matches to known vertebrate NR2 LBDs, with varying degrees of confidence (Table 3). The FAX-1 LBD was most similar to the NR2F2 COUP LBD, albeit with modest significance. The related NHR-111 LBD also matched NR2F2, with much higher significance. The NHR-67 LBD returned a very significant match to the NR2C2 testicular receptor 4 (TR4). In contrast, the NHR-239 sequences did not return significant matches to any structures in the database. Therefore, despite relatively weak sequence conservation between *Caenorhabditis* LBDs and vertebrate LBDs, most NR2E *Caenorhabditis* LBDs retain a predicted structural framework that is similar to defined LBDs.

**Table 3:** **Structural predictions for *Caenorhabditis *LBDs by sequence threading**

**LBD**	**Best structural match**	**PDB ID**	**P**
CbNHR-67	*H. sapiens* NR2C2 TR4 LBD	3p0u	1 x 10^-6^
CeNHR-67	*H. sapiens* NR2C2 TR4 LBD	3p0u	9 x 10^-7^
CbFAX-1	*H. sapiens* NR2F2 COUP LBD	3cjw	0.024
CeFAX-1	*H. sapiens* NR2F2 COUP LBD	3cjw	0.003
CeNHR-111	*H. sapiens* NR2F2 COUP LBD	3cjw	1 x 10^-5^
CbNHR-239	No significant matches.	-	> 0.10
CeNHR-239	No significant matches.	-	> 0.05

Probability values (P) and top match for *Caenorhabditis* LBDs based on PsiPred and pGenThreader [52,53]. Additional significant matches to known NR LBD structures were obtained for each protein, however the significance of matches for both FAX-1 LBDs was low. Species abbreviations are given in Figure 1.

### **The *C. elegans *FAX-1 LBD is not required for some functions *in vivo ***

The higher substitution rate of *Caenorhabditis* LBDs raised the possibility that nematode LBDs have broadly diverse functions—that diversifying selection has led to the evolution of highly-specific LBDs. In order to test this possibility, we used well-characterized *fax-1* mutants and rescuing plasmids to serve as a basis for testing the functional requirements of the FAX-1 LBD [24,26]. A null mutation in the *fax-1* gene causes a distinctive movement defect. *C. elegans* normally moves with a smooth sine wave, but *fax-1* mutants are unable to generate coordinated muscle contractions in their posterior half, leading to severely compromised forward movement. Backing movement is more rapid, but inevitably leads to “coiling”—animals back into a circle on themselves instead of progressing straight backward. These defects may be due to underlying defects in the differentiation of the command interneurons that coordinate forward and backward motility [26,54]. *fax-1* mutations affect multiple interneuron types. In the AVK interneuron pair, *fax-1* mutations cause a high-penetrance defect in axon pathfinding: instead of the AVKR and AVKL axons extending on the respective left and right sides of the ventral nerve cord, the axons are misrouted to the dorsal nerve cord, a lateral nerve bundle, or extend as a pair along the right ventral nerve cord [24]. The anatomy of the AVK neurons can be easily evaluated using anti-FMRFamide antisera [55,56]. *fax-1* function in AVK development is presumably a separate function from the *fax-1* function in movement, since the AVK neurons are not known to be required for movement coordination.

**Figure 4:**
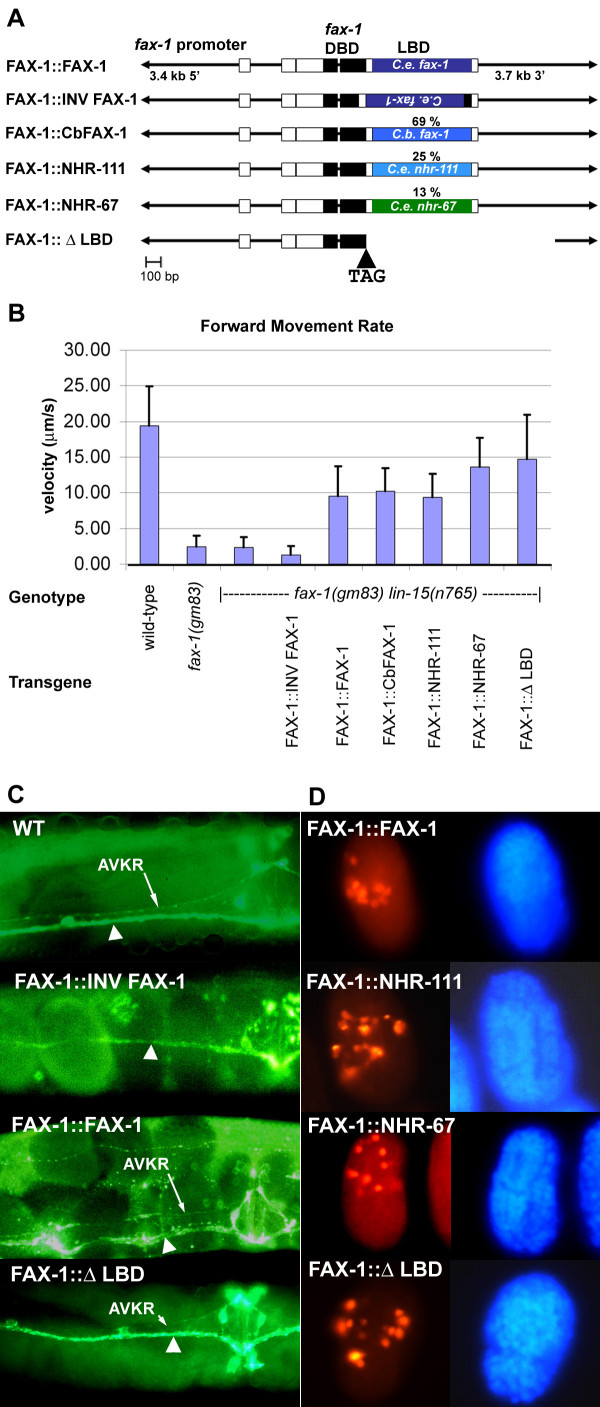
**Evaluation of LBD function for FAX-1.** Design and results of the experiments with LBD swaps among different NR2E LBDs and a deletion of the FAX-1 LBD. **A.** Schematic showing design of the genomic constructs created to test LBD function, as described in Methods. Percentages above LBD boxes indicate the percent identity of the swapped LBD to the *C. elegans* FAX-1 LBD. **B.** Results of movement assays. Figure shows forward movement rates for control wild-type, *fax-1(gm83)fax-1(gm83) lin-15(n765)* and FAX-1::INV FAX-1 strains, as well as each swap construct transgene. **C.** Rescue of the AVKR pathfinding defect in *fax-1(gm83)* mutants by LBD swap transgenes. The wild-type hermaphrodite shows a prominent single AVKR axon located in its proper position in the left bundle of the ventral nerve cord [56]. In the FAX-1:: INV FAX-1 negative control, the AVKR axon is missing from the ventral nerve cord due to misrouting [24]. The FAX-1::FAX-1 and FAX-1:: Δ LBD transgenic animals show the rescued wild-type anatomy. Rescue by FAX-1::Cb FAX-1, FAX-1::NHR-111, and FAX-1::NHR-67 transgenes was equivalent to the examples shown. The circular circuitry of FMRFamide-positive axons around the vulva is at the right side of each figure. All views are ventral views. **D.** Immunofluorescence demonstrating expression of fusion and deletion transgenes in *fax-1(gm83) lin-15(n765)* embryos. Left panels show Cy3 fluorescence detecting the FAX-1 DBD, right panels show matching DAPI staining of nuclei. The FAX-1::FAX-1 and FAX-1::Δ LBD embryos are a somewhat earlier stage (“late comma”), as compared to the FAX-1::NHR-111 and FAX-1::NHR-67 embryos (“two-fold elongation”). The anterior side of each embryo is oriented toward the top. Because of movement of elongation-stage embryos within the egg, orientation of each embryos varies.

To test LBD function, we created a family of DNA constructs that would produce fusion proteins that contained the FAX-1 DBD fused to another NR2E LBD, and a protein that would contain only the FAX-1 DBD without any LBD. Plasmids were based on a 9 kb genomic fragment that fully-rescued the *fax-1* movement and AVK axon phenotypes [24]. For each construct, the genomic region between exon 4 and exon 7 was replaced by a cDNA containing a positive control *fax-1* cDNA sequence (FAX-1::FAX-1), a negative control *fax-1* cDNA sequence in inverted orientation (FAX-1::INV FAX-1), a deleted version of the *fax-1* LBD (FAX-1::ΔLBD) or a cDNA sequence from the *C. briggsae fax-1, nhr-111,* or *nhr-67* LBD (FAX-1::CbFAX-1, FAX-1::NHR-111, or FAX-1::NHR-67; Figure 4A). This strategy created plasmids that would produce FAX-1 DBD::heterologous LBD translational fusion proteins in which the region coding for the FAX-1 DBD was linked to an LBD-coding segment that began in the "hinge region" that separates DBD and LBD, and which replaced the FAX-1 LBD. The encoded fusion genes and deletion construct were under the transcriptional regulation of the *fax-1* promoter in order to ensure expression of the fusion or deleted protein at the correct time and in the correct cells for *fax-1* function.

In order to test the ability of the fusion and deletion constructs to provide *in vivo fax-1* function, we injected each plasmid into *fax-1(gm83) lin-15(n765ts) C. elegans* hermaphrodites, along with a co-transforming plasmid that provides wild-type *lin-15* function. For each transgenic line, we evaluated *fax-1* function by measuring forward movement rates (Figure 4B). While wild-type worms had a forward movement rate of 19.4 (±5.6, N = 10) μm/sec, *fax-1(gm83)* mutants, *fax-1(gm83)* mutants transformed with the *lin-15*- rescuing plasmid alone, and *fax-1(gm83)* mutants transformed with the FAX-1::INV FAX-1 negative control had forward movement rates of 2.40 (s.d. 1.6, N = 10), 2.34 (s.d. 1.5, N = 10), and 1.23 (s.d. 1.3, N = 10) μm/sec, respectively (differences among speeds for negative controls were not statistically significant). The FAX-1::FAX-1 positive control provided a significantly rescued (p < 0.00001) forward movement of 9.6 (s.d. 4.2, N = 14) μm/sec. When the *fax-1* DBD region was fused to another *Caenorhabditis* LBD, we obtained significant rescue (p < 0.00001) of the *fax-1* movement phenotype: forward movement rates ranged from 9.33 (s.d. 3.4, N = 10) μm/sec (for FAX-1::NHR-111) to 13.7 (s.d. 4.0, N = 10) μm/sec (for FAX-1::NHR-67). Likewise, the FAX-1::Δ LBD construct, which contains no LBD at all, was able to provide a robust forward movement rate of 14.7 (s.d. 6.2, N = 17) μm/sec. Differences between *C. elegans* FAX-1, *C. briggsae* FAX-1, and NHR-111 fusion pairs were not statistically significant (p > 0.5), but the increased rescue with the NHR-67 LBD or Δ LBD constructs as compared to other LBDs was significant (p = 0.008 to 0.05). The reason for the improved rescue by the NHR-67 LBD and Δ LBD constructs is not clear. It may reflect a larger number of copies in the transgene, array-specific variation in transcription levels, or structural differences at the level of protein fusion. Therefore, despite a conserved LBD structure and ligand-binding signature region, the FAX-1 LBD is not required for *fax-1* function in the development of neurons that control movement.

We also examined rescue of the *fax-1* phenotype at the level of AVK axonal anatomy [24,56] by the heterologous and deletion LBD constructs. Negative controls displayed a high rate of AVK pathfinding defects in which the AVKR axon was absent from the right ventral nerve cord bundle, while transgenic animals bearing LBD fusions displayed rescue of the AVK pathfinding defect consistent with rescue of the movement defect (Figure 4C; Table 4). In the *lin-15(n765ts)* background, 97% of *fax-1(gm83)* displayed the AVKR axonal defect, an increase from the 78% of *fax-1(gm83)* mutants that showed this defect. *lin-15* functions in vulval development, and this enhancement may reflect the role of vulval precursor cells in organizing the normal bundling arrangement of the ventral nerve cord [57]. The presence of ectopic developing vulvae along the ventral mid-line of *lin-15* mutant animals may increase the severity of the AVKR axonal defect by contributing post-outgrowth bundling defects during larval development. The FAX-1::INV FAX-1 negative control displayed no rescue of the AVKR axon defect, but LBD fusion constructs with *C elegans* FAX-1, *C. briggsae* FAX-1, NHR-111, and NHR-67 all displayed significant rescue of the AVKR axon defect, reducing the penetrance of the defect to between 33% and 61% (Figure. 4C; Table 4). As was the case for the movement defect, rescue with FAX-1::NHR-111 was not as strong as with other *Caenorhabditis* LBDs. Therefore, at the level of axonal anatomy, different *Caenorhabditis* LBDs and a deletion of the LBD were able to provide FAX-1 function.

**Table 4:** Rescue of AVKR pathfinding defects

**TRANSGENE**	**GENOTYPE**	**% AVKR DEFECT**	**N**	**p**
none	wild-type	0	27	
none	*fax-1 (gm83)*	78	32	
*fax-1 9 kb* genomic DNA	*fax-1 (gm83)*	12	25	
none	*fax-1 (gm83) lin-15 (n765)*	97	35	
FAX-1::INV FAX-1	*fax-1 (gm83) lin-15 (n765)*	92	53	0.55
FAX-1::FAX-1 LBD	*fax-1 (gm83) lin-15 (n765)*	33	52	<0.001
FAX-1::CbFAX-1 LBD	*fax-1 (gm83) lin-15 (n765)*	36	22	<0.001
FAX-1::NHR-111 LBD	*fax-1 (gm83) lin-15 (n765)*	61	95	<0.001
FAX-1::NHR-67 LBD	*fax-1 (gm83) lin-15 (n765)*	41	39	<0.001
FAX-1::Δ LBD	*fax-1 (gm83) lin-15 (n765)*	62	63	<0.001

The percentage of the AVKR axon crossover defect [56] is shown for each strain. N = number of AVKR axons examined. p = Chi square P values relative to * fax-1(gm83) lin-15(n756) * strain without any transgene.

We confirmed expression and subcellular localization of our fusion and deletion products by immunofluorescence staining. A mouse anti-FAX-1 antiserum raised to full-length FAX-1 protein detected a strong signal in * fax-1(gm83) lin-15(n765) * embryos that carried a FAX-1 DBD-contained transgene (Figure 4D). Because *fax-1(gm83)* mutants produce no detectable FAX-1 protein [26], the protein detected by the antiserum must reflect expression of the FAX-1 DBD that is common to all fusion and deletion construct transgenes. We detected strong expression by all four fusion transgenes (FAX-1::FAX-1, FAX-1::CbFAX-1, FAX-1::NHR-111, and FAX-1::NHR-67) and the FAX-1::Δ LBD transgene in a spatial and temporal pattern that was nearly identical to the FAX-1 expression pattern in wild-type embryos [26], indicating that our constructs produced stable protein at the correct time and place (Figure 4D; data not shown). The FAX-1::Δ LBD, FAX-1::FAX-1, FAX-1::CbFAX-1 and FAX-1::NHR-67 constructs also showed excellent nuclear localization, suggesting that the LBD is not required for import of FAX-1 protein to the nucleus. The FAX-1::NHR-111 construct was expressed at very high levels, but many embryos showed significant accumulation of protein in the cytoplasm (Figure 4D). It is unclear whether this reflects an artefact of high expression levels or a “dominant” effect of the NHR-111 LBD that inhibits nuclear localization of the FAX-1::NHR-111 fusion protein. Despite the nuclear localization issue, the FAX-1::NHR-111 construct still provided functional rescue, although it is possible that the somewhat weaker rescue by this construct may be accounted for by the compromised nuclear localization.

## Discussion

Analysis of LBD function in the *C. elegans* NR2E subfamily demonstrates the functional independence of some *fax-1* functions on the presence or sequence of the LBD. The simplest interpretation of this result is that the key FAX-1 functions that we have assayed require only binding of the DBD moiety to its cognate recognition site. This is consistent with our previous finding that the FAX-1 DBD alone possesses both sequence specificity and homodimerization function [30]. Furthermore, the DBD also appears to possess sufficient transcription regulation activity to confer normal function. In addition, the DBD expressed *in vivo* without an LBD appears to be efficiently localized to the nucleus, indicating that the LBD is not required for efficient localization. Finally, the absence of a requirement for the FAX-1 LBD demonstrates that it is not ligand-dependent—at least for the functions we have tested. The LBD of the *Drosophila* ortholog of *fax-1,* UNF, has been shown to bind heme [58], although there is no known developmental or physiological requirement for heme-binding. If the FAX-1 LBD can also bind heme-related ligands, our results suggest that this binding is not essential for key FAX-1 functions in *C. elegans*.

The independence of *fax-1* functions from the presence or identity of an LBD suggests that the relatively high level of primary sequence divergence of *Caenorhabditis* LBDs may not reflect diversifying selective pressure toward highly specific functional roles. Instead, the relatively high sequence divergence may result from a release of positive selection on the primary sequence, with sequence differences reflecting drift. Nonetheless, protein sequence threading suggests that some general aspects of secondary and tertiary LBD structure may be maintained in *Caenorhabditis*. Changes in selective pressure on nematode LBDs may have allowed a greater degree of tolerance for substitution within the context of conserved structure.

Analysis of *C. elegans* LBDs and the *Caenorhabditis* genomes has cast doubt on the hypothesis that *C. elegans* NRs mediate transcription using the same components as vertebrate systems. For example, the LBD of several vertebrate nuclear receptors have been shown to bind a common set of coactivators and corepressors, which in turn mediate the effect of the nuclear receptor on transcription [59]. However, an AF2 domain, which is contained within the many vertebrate LBDs and responsible for interaction with p160 coactivators, is absent from most *Caenorhabditis* NRs, including the *fax-1* and *nhr-239* orthologs in this study, and most of the known vertebrate NR coactivator and corepressor genes were absent from a survey of predicted genes in *C. elegans*[9]. Therefore, significant differences between vertebrates and *Caenorhabditis* in the mechanisms of transcriptional control may account for the apparent differences in LBD function in *C. elegans*.

The lack of a requirement for LBD function for *fax-1*rescue in our assay does not necessarily indicate that the FAX-1 LBD provides no function or that *C. elegans* NR2E proteins in general are ligand-independent. On the contrary, the structural conservation of the *C. elegans* LBDs (Table 3), weak purifying selection on LBD sequences (Figure 1), and the general absence of truncated LBDs among conserved NRs in the *C. elegans* genome (save perhaps *nhr-239*), combine to argue that LBDs have been retained because they provide function. It is possible that the FAX-1 LBD is required for subtle aspects of developmental control that are not revealed by our rescue assay. Alternatively, the developmental functions that we have tested could be entirely LBD-independent, but other functions are LBD-dependent. This might be the case if FAX-1 provides an unknown physiological, ecological, or behavioural function during larval and/or adult stages. A precedent for both ligand-dependent and ligand-independent functions exists for vertebrate steroid NRs [60]. It is worth noting that all the *C. elegans* NR2E genes in this study are expressed in larval and adult cells (Figure 3, [26,61], which certainly allows for later functions in addition to the major embryonic developmental functions.

Our preliminary analysis of expression of *nhr-111* and *nhr-239* reveals some commonalities among members of the NR2E family in *Caenorhabditis*. First, all NR2E family members are expressed in subsets of neurons. In the two best studied cases, *fax-1* and *nhr-67*, they play important roles in neuron differentiation and specificity [26,61], functions that are also maintained in flies and vertebrates [23,27,62]. While we have not yet determined functions for *nhr-111* and *nhr-239*, the expression of both genes in subsets of neurons at a time in embryogenesis when neuronal specification is occurring suggests that they may also function in neuronal development. Furthermore, a genomic study focusing on neuron-specific transcriptional complexes identified 15 candidate gene targets for NHR-111*,* including the well-studied neuronal developmental control genes *unc-30* and *unc-86*[63]. In addition, both NHR-111 and NHR-67 were found to bind a common target promoter, suggesting that they may be co-ordinately involved in regulating overlapping neuron-specific genes. A second feature common to all *Caenorhabditis* NR2E genes, except for *nhr-239*, is that they are expressed in the somatic gonad: *fax-1* in the distal tip cells during larval gonadogenesis [24], *nhr-67* in the larval ventral uterine cells, anchor cell, and linker cell of the male [64,65] and *nhr-111* in the Z1 and Z4 gonad precursors and their descendants. Of these, only *nhr-67* has a clearly defined function in uterine development. Nonetheless, the nexus of NR2E expression in somatic gonadal tissues raises the possibility for combinatoric functions within the NR family. At the very least *nhr-111* expression overlaps with *nhr-67* expression in the ventral uterine lineages and anchor cell, and with *fax-1* expression in the distal tip cells. The identification of common targets for both NHR-67 and NHR-111 supports the notion that these transcription factors may have overlapping functions. Arda et al. [66] have identified metabolic gene regulatory networks that are highly-enriched with NRs, both as components of transcription factor networks that regulate genes involved in metabolism, homeostasis, and environmental response, and as targets of regulation by NRs and other transcription factors. Both NHR-67 and NHR-111 were components of modules implicated in coordinated regulation of metabolic genes, similar to what was found with neuron-specific target genes. NHR-111 was also implicated in six additional smaller regulatory complexes, consistent with the relatively broad expression pattern we describe here.

The *C. elegans* NR2E genes that have been studied most thoroughly play various roles in development. Deletion of *nhr-67* results in early developmental arrest [65]. Loss of *fax-1* causes significant movement and nervous system defects, but does not cause lethality [24]. Like *nhr-67, nhr-111* is expressed fairly broadly, but its deletion does not cause lethality or obvious morphological phenotypes (KW, GMB, SC, and BW, unpublished observations). This observation raises the possibility that NHR-111 may partner with other transcription factors in a highly-redundant and overlapping manner to fine-tune gene regulation in many cells. The observation that NHR-111 is a major node in the *C. elegans* interactome map of predicted protein interactions, partnering with itself (suggesting possible homodimerization) and 53 other proteins [67], many of which are potential transcription factors, provides some support for this hypothesis.

Finally, our phylogenetic analysis using a larger set of NR2E sequences from many species argues for three significant evolutionarily-conserved clades: TLL/TLX, FAX-1/PNR, and NHR-239/Hr83. Our data call into question the hypothesis that FAX-1 and PNR represent different genes in the urbilateralian ancestor followed by subsequent loss of PNR in nematodes and loss of FAX-1 in vertebrates [32]. Not only do our phylogenetic data support an orthologous evolutionary origin for FAX-1 and PNR, direct examination of DBD alignments identify consistent conserved loci within each clade, a situation that is less likely to exist due to convergent evolution. A more parsimonious interpretation is a single urbilateralian ancestor for both PNR and FAX-1. To date, we know of no genome that includes a PNR ortholog, a FAX-1 ortholog and an NHR-239/Hr83 ortholog. Instead most animal genomes boast a single PNR-like gene or a single FAX-1-like gene, with a single NHR-239/Hr83 clade member and a single TLL/TLX clade member. Our interpretations of the relationship of NR2E receptors call into question the utility of the systematic nomenclature system for NRs [17] when applied to divergent *C. elegans* receptors. In this case, a version of long-branch attraction or similar artefact has resulted in a confusing situation in which *C. elegans* FAX-1 and insect Hr83 both have been assigned NR2E5 even though FAX-1 is clearly more similar to insect NR2E3 and *C. elegans* NHR-239 is more similar to Hr83. This change in the evolutionary assumptions of the NR2E subfamily does not change predictions of the original number of NRs as described by Bertrand et al., [32], since the “new” NHR-239/Hr83 clade effectively replaces the “lost” FAX-1 clade that results from fusing FAX-1 and PNR into a single clade. In this case, a larger data set was important for drawing the clearest evolutionary inferences. Therefore, complex evolutionary patterns in large gene families may benefit substantially from large-scale sequencing projects that examine closely-related species, such as the current 959 Nematode Genomes project (http://www.nematodes.org).

## Conclusions

We define three conserved clades of NR2E receptors, only two of which are represented in vertebrates. This observation suggests that there were three ancestral NR2E genes in the urbilateria. Additional genes have spawned from descendants of these three ancestral genes, including *nhr-111*, which is a broadly-expressed paralog that appears to have arisen within the *Caenorhabditis* evolutionary lineage. LBD function is not required for at least some important developmental functions of one NR2E family member. This result suggests that the relatively high level of sequence divergence for *Caenorhabditis* LBDs reflects relaxed selection on the primary sequence, rather than highly diversifying positive selection.

## Methods

### **Phylogenetics and computational analysis**

We aligned protein sequences using the alignment utility of MEGA 5.0 [36]. Alignments were performed with the Clustal W algorithm [37] using a BLOSUM matrix, a pairwise gap penalty of 10, with extension penalty 0.1, and a multiple alignment gap penalty of 10, with extension penalty 0.2. We developed phylogenetic trees using the Maximum Likelihood method based on the Dayhoff matrix based model [47] and by Neighbor-Joining [68] using MEGA 5.0 software. All positions containing gaps were eliminated. The bootstrap consensus trees were inferred from 500 replicates. Trees were rooted manually to the *Homo sapiens* RAR gamma outgroup sequence. The trees were drawn to scale, with branch lengths measured in the number of substitutions per site. We evaluated LBD structures of NR2E proteins using PSIPRED and the threading utility pGenTHREADER [52,53] at the Bloomsbury Centre for Bioinformatics at University College London [69]. We calculated Ka/Ks ratios for DBD and LBD regions separately by generating matched Maximum Likelihood trees based on amino acid alignments and nucleotide alignments using Clustal W and MEGA 5.0 as described above. Not all sequences shown in Figure 1 and Additional file 1: Figure S1 were included in the Ka/Ks analysis. Newick-formated trees were generated with MEGA 5.0 and edited by hand to create binary files. Ka/Ks ratios were calculated using the Ka/Ks utility at the Bergen Center for Computational Science, University of Bergen (http://www.bccs.uni.no/units/cbu/).

### **Nematode strains and GFP reporter analysis**

*C. elegans* were cultured as described by Brenner [70] and Stiernagle [71]. Strains were obtained from the *Caenorhabditis* Genetics Center, the National Bioresource Project of Japan, the *C. elegans* Gene Knockout Consortium. *nhr-111::gfp* and *nhr-239::gfp* constructs were created as illustrated in Figure 4. The *nhr-111::gfp* plasmid, pG3.9GFP1, was constructed by amplifying a 2.7 kb region from genomic cosmid clone F44G3 using oligonucleotides [Additional file 3: Table S1] and ligating the product to the GFP expression vector pPD95.79 using BamHI and SphI. The resulting plasmid fused the entire 5’ flanking region of *nhr-111,* 1.2 kb from the predicted start codon to the predicted 3’ end of the immediately adjacent upstream gene, and the genomic *nhr-111* coding region into exon 5 to the coding sequence for GFP. The *nhr-239::gfp* plasmid, pNHR239GFP1, was constructed by amplifying a 2.8 kb region from wild-type *C. elegans* genomic DNA using oligonucleotides [Additional file 3: Table S1] and ligating the product to pPD95.79 using XbaI and XmaI. The resulting plasmid fused 2.1 kb of 5’ flanking DNA, which includes the last intron and exon of the adjacent upstream *feh-1* gene, and the first three predicted exons of the *nhr-239* gene to the coding sequence for GFP. We introduced plasmid constructs into nematodes following standard microinjection techniques [72] using a Nikon UD Optiphot 2 microscope. Transgene-positive progeny were identified by the Roller phenotype conferred by the pRF4 co-transforming marker that bears a dominant mutant version of the *rol-6* gene. We employed a Nikon Eclipse TE 2000 U inverted microscope to examine nematodes using DIC Nomarski microscopy and captured images using a Nikon DMX 1200 camera. To examine and record GFP fluorescence patterns, we used a Nikon UD Optiphot 2 microscope and captured images using a Nikon DS camera. We cropped images and adjusted for optimum contrast and brightness using Adobe Photoshop software.

### **Quantitative real-time qPCR**

We used quantitative real-time PCR (qRT-PCR) [73] to estimate relative levels of *nhr-111* and *nhr-239* expression. Taqman probe/primer mixtures were purchased from Applied Biosystems/Life Technologies (Carlsbad, CA). For both *nhr-111* and *nhr-239*, manufacturer-designed probes were used. In addition, we designed a confirmatory probe/primer mixture for *nhr-239* using Applied Biosystems Primer Express 3.0 software. We prepared staged worm preparations for wild-type *C. elegans* nematodes as follows: embryos, treatment with 20% chlorine bleach and 0.1 M NaOH; L1, bleach treatment followed by overnight incubation in M9 buffer; L2/L3, as for L1 followed by 24 hour feeding on OP50 bacteria at 20°C; L4, as for L1 followed by 48 hour feeding on OP50 bacteria at 20°C. Samples were washed in M9 exhaustively and frozen in RNALater solution (Ambion, Austin, TX). Total RNA was prepared using a RiboPure Yeast RNA kit (Ambion) as directed by the manufacturer. We synthesized cDNA using a High-Capacity cDNA Reverse Transcription Kit (Applied Biosystems) as directed by the manufacturer. Real-time qRT-PCR was conducted on a StepOnePlus system (Applied Biosystems) using Fast Taqman Master Mix (Applied Biosystems). All samples were tested in triplicate or quadruplicate. We measured expression levels in arbitrary units calculated as 2^-ΔCt^x10^10^ relative to 18 S rRNA controls (18 S Eukaryotic Taqman Probe/Primer mix, Applied Biosystems).

### **LBD swap and deletion experiments**

Plasmid pFXCDSp.8 was constructed by cloning an 809 bp SphI fragment from a pFXCD5 *fax-1* cDNA clone into vector pUC18, creating a basic *fax-1* cDNA backbone clone for the purpose of replacing the *fax-1* LBD coding region with cDNA for LBD coding regions of other NRs. To produce FAX-1::FAX-1 and FAX-1::INV FAX-1 constructs, we ligated the 809 bp SphI fragment from pFXCDSp.8 into genomic clone pF56SH9 [24] digested with SphI, thereby replacing the *fax-1* genomic region with a *fax-1* cDNA in the correct (FAX-1::FAX-1) or inverted (FAX-1::INV FAX-1) orientation. To produce FAX-1::ΔLBD, we digested the parent rescuing plasmid pF56SH9 with EcoNI and EagI (which deletes the entire LBD and a portion of the DBD), following by amplification of the 3’ coding portion of the *fax-1* DBD from a *fax-1* cDNA clone using EcoNI and EagI- tailed oligonucleotides (Additional file 3: Table S1) and reconstitution of the intact DBD by ligation of the amplification product into the deleted pF56SH9 plasmid. In addition to a deletion of the LBD, this construct also deleted 468 bp of 3’ UTR and flanking DNA. The resulting FAX-1::ΔLBD plasmid construct was sequenced across the amplified region to confirm wild-type sequence and deletion of the LBD coding region. The LBD swap constructs were created by amplifying the corresponding LBD coding regions from *C. briggsae fax-1, nhr-67,* and *nhr-111,* using corresponding cDNA clones as template, and ligating each to vector pGEM-T Easy using oligonucleotides tailed with BclI, BamHI and/or EcoRI recognition sites (Additional file 3: Table S1)*.* Each LBD cDNA construct was sequenced on both strands to confirm wild-type sequence. We created cDNA fusions for each swap construct by excising the target LBD cDNA with either BclI and EcoRI or BamHI and EcoRI, and ligating the fragment to BamHI/EcoRI-digested pFXCDSp.8. The resulting family of clones created FAX-1::swap LBD cDNA fusion clones with flanking SphI sites. Each cDNA fusion clone was digested with SphI and ligated to the SphI-digested *fax-1* genomic pF56SH9 clone in the proper orientation to create a family of genomic clones that contained replaced LBD cDNA regions. We used a *lin-15* marker for transformation experiments since the more commonly-used *Rol-6* marker would interfere with subsequent movement assays. Each plasmid construct (at 50 μg/ml) and the *lin-15-*rescuing marker plasmid pSK1 (at 50 μg/ml), was microinjected into *fax-1(gm83) lin-15(n765ts)* hermaphrodites that had been grown at 15°C. The *lin-15* mutation results in a multi-vulva (Muv) phenotype at the non-permissive temperature of 25°C, but is near wild-type when grown at 15°C. Injected hermaphrodites were grown at 25°C to allow identification of transformed non-Muv animals in the next generation. Each transgene was maintained as one or more independent extrachromosomal arrays by picking wild-type animals at 25°C. For each construct we obtained at least two dozen transient F1 non-Muv transformants and established between two and twenty independent stable lines. Transgenic lines and control strains were evaluated for movement using a Nikon SMZ800 stereo dissection microscope outfitted with a Hitachi CCD camera KP-D20BU. Video was captured for 30 seconds per trial using Mitotic Images Plus software. Worm movement was measured by digital calibration of distance moved per second during bursts of forward movement. Some *fax-1(gm83)* and non-rescued transgenic strains made no forward progress during the trial period, which was scored as 0 μm of movement over the period of apparent attempted movement. We calculated forward movement speeds as μm/sec. The AVKR axon pathfinding phenotype of transgenic and control strains was evaluated using immunofluorescence and an anti-FMRFamide antibody as described previously [24,56]. We evaluated expression of deleted and fusion proteins from each transgene using immunofluorescence and a mouse anti-FAX-1 polyclonal antibody, and a Cy3-labeled Goat anti-mouse IgG secondary antibody as described previously [26]. The anti-FAX-1 antibody was raised to full-length FAX-1 protein and was able to detect transgene-expressed protein from each fusion construct and the deletion, indicating that the antiserum contains antibodies that recognize the DBD. Different transgenic lines containing the same construct differed somewhat in the rate at which the array transgene was transmitted, but did not differ substantially from each other in rescue or expression experiments. For each construct, the data reported here are from one representative transgenic strain.

## Competing interests

The authors declare that they have no competing interests.

## Authors’ contributions

KPW constructed the majority of the LBD fusion constructs, evaluated *nhr-111::gfp* expression, performed some of the computational analysis, and edited the manuscript. CGA created and evaluated the *nhr-239::gfp* constructs, performed some of the qRT-PCR analysis, and edited the manuscript. GMB evaluated *nhr-239::gfp* expression and the *nhr-111* mutations. KR created the *nhr-111::gfp* construct. GC contributed to construction of the LBD fusion constructs. SC performed genetic back-crossing, contributed to the LBD fusion constructs, managed some of the experiments, and edited the manuscript. BW conceived the project, drafted the manuscript, performed phylogenetics analysis, constructed the LBD deletion construct, performed the nematode transformations, some of the GFP expression analysis, and some of the qRT-PCR experiments. All authors read and approved the final manuscript.

## Supplementary Material

Additional file 1 **Figure S1.** Phylogenetic analysis of NR2E DBDs. Click here for file

Additional file 2 **Figure S2.** Sequence alignments of NR2E LBDs. Click here for file

Additional file 3 **Table S1.** Oligonucleotide sequences. Click here for file
