# Supplementary material for: Analysis of C. elegans NR2E nuclear receptors defines three conserved clades and ligand-independent functions
Source: BMC Evol Biol. 2012 Jun 12;12:81. doi: 10.1186/1471-2148-12-81 (PMC3517510; doi:10.1186/1471-2148-12-81)
Supplement: Additional file 1 — Figure S1. Phylogenetic analysis of NR2E DBDs. [file 1471-2148-12-81-S1.doc]

| **TABLE S1 Oligonucleotide sequences** | |
| --- | --- |
| Oligo name | Oligo sequence |
|  | *nhr-111::gfp* |
| OF44951 | GAGCATGCTTGAATAAATTTGAGACAGACCGAC |
| OF44931 | GAGGATCCGCAACTCGACATACTCTACAGTGTA |
|  |  |
|  | *nhr-239::gfp* |
| OY54IL | TTTTCAGATTCTAGGCCGTCA |
| OY5431X | CACCCGGGCAACCTTGTGCATTTTGCAA |
|  |  |
|  | LBD swap constructs |
| OCBFXLBD51 | CCGGATCCTCAGAACTTCTTTCGGGAG |
| OCBFXLBD32 | GACCCGGGCATGCGGTTGTGTAGATTGGAGCAAACAAA |
| ON111LBD51 | CCGGATCCTCTGCCTACTCACTCTGATCGTCTC |
| ON111LBD31 | CCGAATTCGCATGCGTGTGCATATAACAGCATCAGAAAA |
| ON67LBD51 | CCTGATCAGACATCATCAATGAATCGTGACACA |
| ON67LBD31 | CCGAATTCGCATGCTAAACCTCTTCAACATTAACACTC |
|  |  |
|  | LBD deletion construct |
| OLBDEL51 | TGCGTTGTAGACAAAGCACATAGAA |
| OLBDEL31 | ATCGGCCGCTATTGTGGATCCATGTCAAGGG |
|  |  |
