# Supplementary material for: Analysis of C. elegans NR2E nuclear receptors defines three conserved clades and ligand-independent functions
Source: BMC Evol Biol. 2012 Jun 12;12:81. doi: 10.1186/1471-2148-12-81 (PMC3517510; doi:10.1186/1471-2148-12-81)
Supplement: Additional file 2 — Figure S2. Sequence alignments of NR2E LBDs. [file 1471-2148-12-81-S2.pdf]

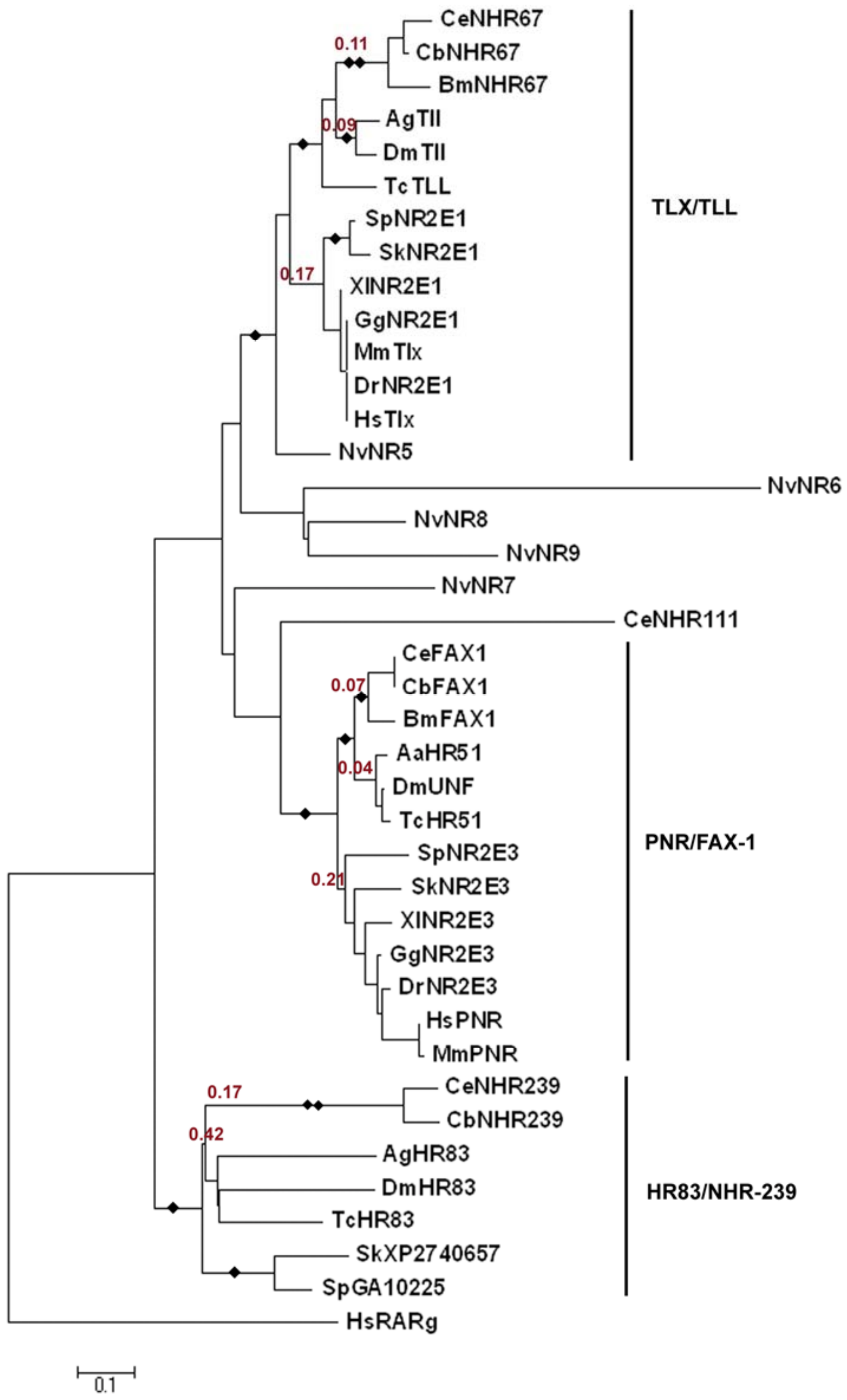
CeNHR67
CbNHR67
BmNHR67
AgTll
DmTll
TcTLL
SpNR2E1
SkNR2E1
XlNR2E1
GgNR2E1
MmTlx
DrNR2E1
HsTlx
NvNR5
NvNR6
NvNR8
NvNR9
NvNR7
CeNHR111
CeFAX1
CbFAX1
BmFAX1
AaHR51
DmUNF
TcHR51
SpNR2E3
SkNR2E3
XlNR2E3
GgNR2E3
DrNR2E3
HsPNR
MmPNR
CeNHR239
CbNHR239
AgHR83
DmHR83
TcHR83
SkXP2740657
SpGA10225
HsRARg
TLX/TLL
PNR/FAX-1
HR83/NHR-239
0.11
0.09
0.17
0.07
0.04
0.21
0.17
0.42
0.1

Fig. S1. Phylogenetic analysis of NR2E DBDs

A consensus tree generated from 500 bootstrap replicates and rooted to the *Homo sapiens* retinoic acid receptor gamma DBD outgroup sequence is shown. Further details are provided in Methods. Species abbreviations (used throughout): Ce (or no annotation)*: Caenorhabditis elegans,* Cb*: Caenorhabditis briggsae,* Bm: *Brugia malayi*, Pp: *Pristionchus pacificus,* Dm: *Drosophila melanogaster*, Ag: *Anopheles gambiae*, Tc: *Tribolium castaneum*, Sk: *Saccoglossus kowalevskii*, Sp: *Strongylocentrotus purpuratus*, Dr: *Danio rerio,* Xl: *Xenopus laevis*, Mm: *Mus musculus,* Gg: *Gallus gallus*, Hs: *Homo sapiens*, Nv: *Nematostella vectensis*, Aa: *Aedes aegypti*. Tree is proportionally scaled, with the scale bar indicating sequence distance in units of substitutions. Single diamonds identify branchpoints that were supported by >50% of bootstrap replicates; double diamonds identify branchpoints that were supported by >95% of bootstrap replicates. Numbers in red above selected branches show Ka/Ks ratios for the DBD portion only of NRs.

Weber et al., 2012
