# Supplementary material for: Analysis of C. elegans NR2E nuclear receptors defines three conserved clades and ligand-independent functions
Source: BMC Evol Biol. 2012 Jun 12;12:81. doi: 10.1186/1471-2148-12-81 (PMC3517510; doi:10.1186/1471-2148-12-81)
Supplement: Additional file 3 — Table S1. Oligonucleotide sequences. [file 1471-2148-12-81-S3.pdf]

```
                                            SIGNATURE
LBD Features                     h   hh ΦAK hP  F   L    DQ   LL        h hh

HsRARg          DKFSELATKCIIKIVEFAKRLPGFFTGLSIADQITLLKAACLDILMLRICTRYTPEQDTMTFSDGLTLNRTQMHNAG---- 76

CbFAX1          DSLQESALSHMESILQWAQQFRLFAVLTESEKRQIILTQWPRLLCIALCEQSEDASLD---------------------- 58
CeFAX1          DSLQETTMSQLESVLQWAQQFRLFTVLTNSEKRQIILTQWPRLLCISLCEQAEDVSFD---------------------- 58
CeNHR111        -----SVLVHLKNALQWVQQFSLFAVLSDVEKSQIILTQWPHLLCLALFENSEKIFID---------------------- 53
BmFAX1          ENVQEASTRLLFLAIKWAKNLPSFASLSLRDQLKLLKENWCDLFLLSVFQWSLPMDK--CPLLNTLQTDPS--------- 69
PpFAX1          -HSHALLRKYAGCAVSAVLSQPDSTSLLPSDEDASPLSEESKLETLRSILAGNAGVCEAPPAP----------------- 62
DmUNF           ETVYETSARLLFMAVKWAKNLPSFARLSFRDQVILLEESWSELFLLNAIQWCIPLDPTGCALFSVAEHCNNLENNANGDT 80
AaHR51          ETIYETSARLLFMAVKWAKNLPSFASLTFRDQVILLEESWSELFLLNAIQWCMPIDTSACTLFSLNEHCSSVNNSG---- 76
TcHR51          ETIYETSARLLFMAVKWAKNLPSFASLPFRDQVILLEEAWSELFLLNAIQWCMPLDVSASPLFNVNEHVKNGHS------ 74
SpNR2E3         DSIYESSARLLFMAVKWAKTLPSFSGLPFRDQVILLEEAWSELFLLCALQWSMPLDS--CPLLTGLHEQSQ---TDKAA- 74
SkNR2E3         ENIYESSARLLFMAVKWAKNLPSFSALPFRDQVILLEEAWSELFLLCAIQWSMPLES--SPLLVAPEQSQATQLNGKTA- 77
DrNR2E3         ESVYETSARLLFMSVKWAKNLPVFSHLPFRDQVILLEEAWSELFLLCAIQWSLPLDN--CPLLSLPDLSPT--GQGKGS- 75
XlNR2E3         EGVYETSARLLLMAVKWAKNLPVFSNLPFRDQVILLEEAWSELFLLCAIQWSMPLDS--CPLLSVPDLSSQ--VHGKSV- 75
GgNR2E3         ENVYETSARLLFMAVKWAKNLPVFSNLPFRDQVILLEEAWSELFLLCAIQWSMPLES--CPLLAVPEPS-----PGKLL- 72
MmPNR           DGIHETSARLLFMAVKWAKNLPVFSNLPFRDQVILLEEAWNELFLLGAIQWSLPLDS--CPLLAPPEASGS--SQGRLA- 75
HsPNR           DSIHETSARLLFMAVKWAKNLPVFSSLPFRDQVILLEEAWSELFLLGAIQWSLPLDS--CPLLAPPEASAAGGAQGRLT- 77

CbNHR67         DSVVGTAARIFFALVGYCQN--ALTGVPREQQLAIFQQHWATLLLLHATESRAITSRQIRNEVTSGTSKLR--------- 69
CeNHR67         DTVAGTAARIFFALVGFCQN--PLNGVPKERQMTMFQQNWAALLVLHATETRAITSKQIRTETISGSSEQR--------- 69
BmNHR67         -TTRDAAARLFFQILHWSKSLIAFACLSPQEQVAAFTSSWGVLFLLSAVENRFLQSS-----VLANERTIN--------- 65
DmTll           EHIKETAAEHLFKNVNWIKSVRAFTELPMPDQLLLLEESWKEFFILAMAQYLMPMNFAQLLFVYESENANREIMG----- 75
AgTll           DAIRESAAQLLFMNVNFLKSLTPFTQLPMADQLVLFEESWREFFILAVAQYLAPINFSQLLIAYEYLNNNRGETGTVSD- 79
TcTLL           SAICESAAQLIFMNVQWVRSIPAFTCLPLSDQLLLLEESWLDLFVLGAAQFLPLMDFSVLVEACGVLQQEPHRRD----- 75
SpNR2E1         DAICETAARLLFMSIRWVKNVPAFIGLPYSDQLTLLEEGWRELFILGAAQWQMTVDG--PGLMASAGMKPD--TTP---- 72
SkNR2E1         EAICETAARLLFMSVKWAKNVPAFLSLPFRDQLLLLEEGWRELFVLGAAQWQMCMEI--GPLLAAAGLSTE--HTN---- 72
DrNR2E1         ESVCESAARLLFMSIKWAKSVPAFSTLPLPDQLILLEDAWRELFVLGIAQWAIPVDS--STLLAVSGMNTE--NTD---- 72
XlNR2E1         ESVCESAARLLFMSIKWAKSVPAFSTLSLQDQLMLLEDAWRELFVLGIAQWAIPVDA--STLLAVSGMNNE--NTE---- 72
GgNR2E1         ESVCESAARLLFMSIKWAKSVPAFSTLSLQDQLMLLEDAWRELFVLGIAQWAIPVDA--NTLLAVSGMNGD--NTD---- 72
MmTlx           ESVCESAARLLFMSIKWAKSVPAFSTLSLQDQLMLLEDAWRELFVLGIAQWAIPVDA--NTLLAVSGMNTD--NTD---- 72
HsTlx           ESVCESAARLLFMSIKWAKSVPAFSTLSLQDQLMLLEDAWRELFVLGIAQWAIPVDA--NTLLAVSGMNGD--NTD---- 72

CbNHR239        -----------------------LRCITIPRINNLNSFKYDMIFSKAVSLTG---------------------------- 29
CeNHR239        -----------------------LRCLQLKHTNNPISFKYDSIFSKSVILTS---------------------------- 29
DmHR83          ALHFQILAQILVTCLRQAKANEQFALLDRCQQDAIFQVVWSEIFVLRASHWSLDISA-------MIDGCGD--------- 64
AgHR83          GLSVQILSQVLMACIRQVRHNEHFAIFSRAQQNEILRHVWYECFLLRVANWSIDISS-------LVERCCD--------- 64
TcHR83          -PIQELAAQILLVAIKQARCNSGFGLLNRASQNLILSHLWAPLFLLRAAHWPSESAD----------------------- 56
SpGA10225       SAIEEAATQIIIHALRTSKSVQPFRALDPWDQNSLLQECWAELFLLHAAYWPP---ADFCALLSHSHLRMDDAKTETDS- 76
SkXP2740657     EMLHEIAAQILFTSIKRARSVQTFQTLSFSDQILLLEDCWGELFLLHAAYWP----VDLATLIVQFQGSADTAAGSG--- 73
```

```
LBD Features                            E   h  h Lh                                        h

HsRARg          ------FGPLTDLVFAFAGQLLPLEMDDTETGLLSAICLICG-----------------------------DRMDLEEPE 121

CbFAX1          -----------EHLGSIMLKFRRLDVSPAEFNCMKAITIFMKRTIG------------------WRSKHHYRIPQTTCVP 109
CeFAX1          -----------DHLTSLMLKFRRLDVSPAEFNCLKAITIFMKRELSL-----------------WRAGWDNRASIITVYP 110
CeNHR111        -----------EKFAQLAEKFKSLELSAQDYFLLKGIIIFTET----------------------------------KDG 88
BmFAX1          ---------SFRYLNDLFFRIRSYGIDHGEFACLKAIVLFRP-----------------------------ETRGLKNLV 111
PpFAX1          ---------------------PVERIVRASFDWARGLPSFAALP--------------------------------KDDQ 89
DmUNF           CITKEELAADVRTLHEIFCKYKAVLVDPAEFACLKAIVLFRP-----------------------------ETRGLKDPA 131
AaHR51          VFKPGQLAQDLRVLNDTLCRFKSVMVDPAEFACMKAIVLFRS-----------------------------EARGLKDPV 127
TcHR51          -------ATDVRILADTLMRFKAIHVDPAEFACLKAIVLFRS-----------------------------ETRGLKDPS 118
SpNR2E3         -----TCVSDIRLLQEIMSRFRGLRVDPAEFACLKAIVLFKP-----------------------------ETRGLKDPQ 120
SkNR2E3         -----AMLSDIRVLQEIMARFKAMNVDPAEFACMKAIVLFKP-----------------------------DTRGLKDPQ 123
DrNR2E3         -----PSASDVRVLQEVFSRFKPLQVDPTEFACLKAIVLFKP-----------------------------ETRGLKDPE 121
XlNR2E3         -----SSTIDVRILQETISRFKSLNVDPTEFACLKAVLLFKP-----------------------------ETRGLKDPE 121
GgNR2E3         -----PAAVDVRALQETLGRFKALAVDPTEFACMKAVVLFKP-----------------------------ETRGLKDPE 118
MmPNR           -----LASAETRFLQETISRFRALAVDPTEFACLKALVLFKP-----------------------------ETRGLKDPE 121
HsPNR           -----LASMETRVLQETISRFRALAVDPTEFACMKALVLFKP-----------------------------ETRGLKDPE 123

CbNHR67         -----------NEVAAAFEMIEGLHLDTREYSLLKIMTLMRA-------------------------------------- 100
CeNHR67         -----------NAVANAFEIIERLQLDNREYMMLKHFTMWRD-------------------------------------- 100
BmNHR67         -------IQMRMKLLSAVSQLEQLRLDSTEYNQLRLLSLIKG-------------------------------------- 100
DmTll           -----MVTREVHAFQEVLNQLCHLNIDSTEYECLRAISLFRKSPPSASSTEDLANSSILTGSGSPNSSASAESRGLLESG 150
AgTll           -----FLVKEVEIFQEILAQLAALRVDPNEYVYLRAIVLYKSEFDAETSISSVSSD----GSDVTTASSAGSAKSIGEIA 150
TcTLL           -----AFLKEVADFQETLKKISQFQLDAHEFACLRAIVLFKTSFEKPS-------------------SSSNQEKTTTESA 131
SpNR2E1         AEKLAAISSELRVLQELIAKFRQLNVDDTEFACLKGIVIFK-----------------------------TDISGIKETS 123
SkNR2E1         PEKIVAIMSEMRTFQEIIAKFKQMQVDATEYACLKGIIIFKS----------------------VFPDSPQEIRGVRDFH 130
DrNR2E1         SQRLNKIISEIQALQEVVTRFRQLRLDATEFACLKCIVTFKA----------------------VPTHSGSELRSFRNAS 130
XlNR2E1         SPKLNKIISEIQALQDVVSRFRQLRLDATEFACLKCIVTFKAG---------------------VSTHSGSELRNFRNAA 131
GgNR2E1         SQKLNKIISEIQALQEVVARFRQLRLDATEFACLKCIVTFKA----------------------VPTHSGSELRSFRNAA 130
MmTlx           SQKLNKIISEIQALQEVVARFRQLRLDATEFACLKCIVTFKA----------------------VPTHSGSELRSFRNAA 130
HsTlx           SQKLNKIISEIQALQEVVARFRQLRLDATEFACLKCIVTFKA----------------------VPTHSGSELRSFRNAA 130

CbNHR239        -------------------------------KCILLNFMISE-------------------------------------Q 41
CeNHR239        -------------------------------KCLLLNFMSQQ-------------------------------------E 41
DmHR83          -----------EQLKRLICEAHQLRADVLELNFMESLILCRK------------------------------ELAINAEY 103
AgHR83          -----------GHLRSVMEDIKALRVDLIELSLLETLILCRK------------------------------EFALSARE 103
TcHR83          ---------LFPGVKGTFRVVRQLKLNLTDLEIVENILLCRA--------------------------------DILDDV 95
SpGA10225       KGATRRKSEVVDDIQEITVRLRTLNLSTHEFAFLEAIVLFKPD---------------------T-------KGTLREKS 128
SkXP2740657     --------------------------------FDSLKVNFKEG--------------------------------LGNTQ 89
```

```
LBD Features     h  h      L                 Φ  L   h  h  h                       AF-2

HsRARg           KVDKLQEPLLEALRLYARRRRPSQPYMFPRMLMKIT-DLRGISTKGAERAITLKMEIPGPMPPLIREMLENPEM 194

CbFAX1           KRDEKRRHDVVALCRRFCRLMGFN-CVIPLALVSFLPKCEMQPRHAINSLPTACVPGGSSAHPELGCSIEP--- 179
CeFAX1           AGERGARLVAAALLLAEHSVMGFGNCVIPLALVFSTKSRYVIQRHAINSLP-ACVPGGTSAHPVLRCSMGS--- 180
CeNHR111         TDLKFDRQLDICIGLLNQLHLESSKSKSGRLLFLLG-ELKSYSTRQLESLLDLKACEIVISFL----------- 150
BmFAX1           QIEDLQDQAQQTLAKHTMNSSPAR---FGRLLLLLP-LLRTISAEKIERMFFMATFGNTSIDQIICKMYNG--- 178
PpFAX1           TALLSSKWTSLYLLHCVEAALGSEKCPALEHICGGS-RENLDRSRVLFSLLSDADRGEIACLKAITLFHNVPQD 162
DmUNF            QIENLQDQAHVMLSQHTKTQFTAQIARFGRLLLMLP-LLRMISSHKIESIYFQRTIGNTPMEKVLCDMYKN--- 201
AaHR51           QIENLQDQAQVMLAQHSRTQFPGQIARFGRLLLMLP-LLRIINSHKIESIYFQKTIGNTPMEKVLCDMYKN--- 197
TcHR51           QIENLQDQAQVMLWQHCRTQLPGQVARFGRLLLMLP-LLRIVPASRVEAVFFQKTIGNTPMEKVLCDMYKN--- 188
SpNR2E3          QVEILQDQAHMMLTQHIRAHQPAQTARFGRMLLLLP-SLRFVTSDQVERLFFRCTIGDTPMERLLCDMFKN--- 190
SkNR2E3          QVENLQDQAQLMLGQHTR-NHPTQPTRFGRLLLMLP-SLRFVTPNRIENLFFHHTIGNTPMERLLCDMFKN--- 192
DrNR2E3          QVENLQDQSQVLLAQHIHTLYPSQVARFGRLLLLLP-SLHFVSSERIEHLFFQRTIGNTPMEKLLCDMFKN--- 191
XlNR2E3          QIENLQDQSQMMLAQHTRNQYPAQPVRFGKLLLLSP-SLRFISSERIELLFFHRTIGNTPMEKLLCDMFKN--- 191
GgNR2E3          QVENLQDQSQVMLGQHNRSHYPGQPVRFGKLLLLLP-ALRFLSSERVELLFFRRTIGNTPMEKLLCDMFKN--- 188
MmPNR            HVEALQDQSQVMLSQHSKAHHPSQPVRFGKLLLLLP-SLRFLTAERIELLFFRKTIGNTPMEKLLCDMFKN--- 191
HsPNR            HVEALQDQSQVMLSQHSKAHHPSQPVRFGKLLLLLP-SLRFITAERIELLFFRKTIGNTPMEKLLCDMFKN--- 193

CbNHR67          --TPNGQQIAYQLMTLQAVTHRTDQLRFWKCYTATT----TTPTSAIIDVLFRPSIGSASMTRLIEDMFKPPKP 168
CeNHR67          --TPSAIQIVFQLASIQNFTHRTEPTRYIQCINAIA----AIPTTSIIDVLFRPSIGSASMPRLIQDMFKPPQQ 168
BmNHR67          RNPQMEQLTAFNFAQHQQITYPCQPLRYISCMVLLE---TMPQEAILSELYFKRSIGNASMSALVADILLPKDL 171
DmTll            KVAAMHNDARSALHNYIQRTHPSQPMRF-QTLLGVVQLMHKVSSFTIEELFFRKTIGDITIVRLISDMYSQRKI 223
AgTll            TVRALEESAKEALASYISTCRPGPSNRY-RTLLQLLPALRNVSSYTIEELFFRRNIGPAPLLKLLLDFYRQK-- 221
TcTLL            KISVIQDDAQMRLNKHVTTTYPKQPLRFGKILLLVSSTFRTISGRTIEDLFFKKVIRDTPIVAIISNMYKNQIL 205
SpNR2E1          SVVTLQDQSQLALSKYITVRHQTQPYRFGKLLLLLP-SVRAIRPTTLEQIFFWKAVGSTPFHTLLTDLYKKNEH 196
SkNR2E1          GVATLQDQAQLTLSKYIHTKYPTQPFRFGKLLLMLP-QLRAIRPSTIEELFFRKTIGNIPIERLLCDMYKANDF 203
DrNR2E1          AIAALQDEAQLTLNSYIHTRYPTQPCRFGKLLLLLP-ALRSVGPSTIEEVFFKKTIGNVPITRLLSDMYKSSDI 203
XlNR2E1          AISALQDEAQLTLNSYIHTRYPTQPCRFGKLLLLLP-ALRSINPSTIEEVFFKKTIGNVPITRVLSDMYKSSDI 204
GgNR2E1          AIAALQDEAQLTLNSYIHTRYPTQPCRFGKLLLLLP-ALRSISPSTIEEVFFKKTIGNVPITRLLSDMYKSSDI 203
MmTlx            AIAALQDEAQLTLNSYIHTRYPTQPCRFGKLLLLLP-ALRSISPSTIEEVFFKKTIGNVPITRLLSDMYKSSDI 203
HsTlx            AIAALQDEAQLTLNSYIHTRYPTQPCRFGKLLLLLP-ALRSISPSTIEEVFFKKTIGNVPITRLLSDMYKSSDI 203

CbNHR239         RREVVEENCQMIFALLLLLNGDQTTIRRFTTNENVP---SGVSCDPEQLRLLVCIILSQSSEFQ---------- 102
CeNHR239         RREVVEENCQIIFALLILASGDEAVLSRFPVSFWVS---QRILP------------------------------ 82
DmHR83           AVILGSHSKAALISLARYTLQQSNYLRFGQLLLGLRQLCLRRFDCALSCMFRSVVRDILKTL------------ 165
AgHR83           ATQLEQFAERALVALEP-TAALPAPARLGKLLLGLRTVALRFNEFAVRAMLREVISDEGLALETILTKL----- 171
TcHR83           EQITLALNVLTRALDELAVRTVLERRRFADILLALP-VLFVPSAVVLHSLLFKPVIGAVPIETVISTI------ 162
SpGA10225        KVEFFRDQSQVVLAQYENIVHPESPARFGKLLLTMP-ALKRVGTENLEELFFRRTLGKVQIEKILERM------ 195
SkXP2740657      QVEFLQDQAQLILAQYVNSKTPQNPARFGKLLLTLA-SLRTYKSEIIEELFFRKTIGKVPIEAIFGSV------ 156
```

FIGURE S2. Alignments of LBD regions of NR2E proteins.
Multiple alignments were performed using CLUSTAL W using a BLOSUM matrix and a pairwise gap penalty of 10 with 0.1 extension penalty and a multiple alignment gap penalty of 10 with 0.2 extension penalty (Thompson et al., 1994). Numbers at right margin identify amino acid positions relative to the first residue after the DBD. The alignment was colorized using the Color Align utility of the Sequence Manipulation Suite (Stothard, 2000; http://www.bioinformatics.org/). Key LBD features were annotated following the analysis of critical conserved LBD features from structures of vertebrate LBDs (Wurtz et al., 1996).

Weber et al., 2012.

Key:

G, A, V, L, I
F, Y, W
C, M
S, T
K, R, H
D, E, N, Q
P

REFERENCES:

Stothard P., 2000, The Sequence Manipulation Suite: JavaScript programs for analyzing and formatting protein and DNA sequences. Biotechniques 28:1102-1104.

Thompson JD, Higgins DG, Gibson TJ., 1994, CLUSTAL W: improving the sensitivity of progressive multiple sequence alignment through sequence weighting, position-specific gap penalties and weight matrix choice. Nucleic Acids Res., 22: 4673-4680.

Wurtz JM, Bourguet W, Renaud JP, Vivat V, Chambon P, Moras D, Gronemeyer H., 1996, A canonical structure for the ligand-binding domain of nuclear receptors. Nat Struct Biol. 3(1):87-94.
